# TGF-β3 regulates adhesion formation through the JNK/c-Jun pathway during flexor tendon healing

**DOI:** 10.1186/s12891-021-04691-x

**Published:** 2021-09-30

**Authors:** Ke Jiang, Yuling Li, Chao Xiang, Yan Xiong, Jiameng Jia

**Affiliations:** 1 Department of Orthopaedics, Affilliated Hospital of North Sichuan Medical College, 63 Wenhua Road, Nanchong, Sichuan 637000 People’s Republic of China; 2 Key Laboratory of Emergency and Trauma, Ministry of Education, College of Emergency and Trauma, Hainan Medical University, Haikou, 571199 People’s Republic of China; 3 Department of Orthopaedics, Daping Hospital, Army Medical University, Chongqing, 400042 People’s Republic of China; 4 Department of Rehabilitation, Affilliated Hospital of North Sichuan Medical College, Nanchong, Sichuan 637000 People’s Republic of China

**Keywords:** Adhesion formation, Flexor tendon, TGF-β3, Repair

## Abstract

**Background:**

The injured flexor tendon has poor healing ability, which is easy to cause tendon adhesion. It can affect the recovery of tendon function, which is still a long-term and difficult task for surgeons. Transforming growth factor β (TGF-β) has been widely considered to play an important role in flexor tendon repair in recent years.

**Aim:**

This work was to investigate the anti-adhesion and anti-inflammatory effects of TGF-β3 on flexor digitorum longus (FDL) tendon repair rats.

**Method:**

Anastomosis models of tendon laceration in the flexion toes of rats were delivered with no treatment, vehicle, or TGF-β3 -overexpressed adenovirus vector (ad-TGF-β3) locally to the injured tendon area from day 3 to 8. Subsequently, the expression of TGF-β3, TGF-β1/2, Smad3, Smad7, JNK, phosphorylation (p)-JNK, c-Jun, and phosphorylation (p)-c-Jun were detected by western blot, the expression of Mmp9 and Mmp2 by RT-qPCR, the Range of motion (ROM) and gliding resistance by adhesion formation testing, the mechanical strength of tendon healing by biomechanical testing, the pathologic changes of flexor tendon tissues by HE staining, the expression of collagen type III by immunohistochemical staining, and the levels of IL-6, TNF-α, COX2 and IL-1β in serum by ELISA, respectively.

**Results:**

Rat models treated with no treatment showed a lower elevation of TGF-β3 and Smad7 expression, and a higher elevation of TGF-β1/2 and Smad3 expression, during day 14 to day 28. Besides, under the treatment of ad-TGF-β3, a significantly increase was reflected in the expression of TGF-β3 and Smad7, ROM, as well as mechanical strength of flexor tendon, whereas significantly reduction was shown in gliding resistance, the content of inflammatory cytokines, the ratio of p-JNK/JNK, p-c-Jun/c-Jun, as well as the expression of TGF-β1/2, Smad3, Mmp9, and Mmp2 genes, as compared to those from vehicle treatment. Meanwhile, TGF-β3 demonstrated a better pathologic recovery process with no obvious necrosis or fracture of collagen fibers. Besides, TGF-β3 revealed a significant reduction of collagen type-III expression in the flexor tendon healing tissues.

**Conclusion:**

These findings suggested that TGF-β3 effectively protected against flexor tendon injury via regulating adhesion formation.

## Introduction

The injured flexor tendon had poor healing ability, which was easy to cause tendon adhesion. It could affect the recovery of tendon function, which was still a long-term and difficult task for surgeons [1, 2]. After tendon trauma, the synovial cells of the tendon sheath were damaged with secondary inflammatory reactions [3]. Once the tendon sheath synovial cells were damaged, the macrophages, neutrophils, and other cells released a variety of cytokines such as Mmp9 and TNF-α, which will stimulate the proliferation of peripheral fibroblasts, inhibiting the growth of tendon cells and matrix synthesis, and causing irreversible apoptosis in tendon sheath synovium cells [3]. Though the proliferation of fibroblasts and the synthesis of matrix would restore the continuity and strength of tendon fibers and promote tendon healing, it would cause the formation of fibrous scar tissue and adhesion as well, which lead to the loss of postoperative tendon slippage and finger dysfunction.

Transforming growth factor β (TGF-β) was a multifunctional family of polypeptide cytokines that played an important role in regulating cell proliferation, differentiation, ECM component metabolism, tissue injury healing, and organ fibrosis. Previous studies had shown that TGF-β regulated a wide variety of cells involved in tissue repair, and such factors were considered key to tendon scarring and adhesion during tendon healing [4]. TGF-β1 and β2 mediated tissue fibrosis and scarring [5, 6], with TGF-β3 acting as an inhibitor of these events [7]. Foreign studies had reported that TGF-β3 was believed to have the dual effects of antagonizing TGF-β1 and improving post-traumatic scar formation [8, 9]. In the scarless healing process after tendon injury in sheep embryos, expression of TGF-β1 was decreased, while TGF-β1 expression was increased in the healing process after tendon injury in adult sheep [10]. In the development of chicken embryo tendon, TGF-β1 expression was absent in the development of the tertiary tendon tract on days 13, 14 and, 15, and TGF-β3 expression shows a dynamic change [11]. However, the molecular mechanism of TGF-β3 therapy, especially the anti-adhesion and anti-inflammatory response during flexor tendon healing, was still unclear.

In the preliminary experiment, we studied the regulation of Smad3 and Smad7 proteins in the TGF-β/Smad signaling pathway of tendon cells [12]. It revealed that TGF-β3 down-regulated the expression of Smad3 protein and up-regulated the expression of Smad7 protein in the process of tendon cell injury healing, which preliminarily elucidated the possible regulatory mechanism of TGF-β3 in promoting scar-free tendon healing [12], and may therefore be an effective treatment in anti-adhesion formation.

As a serine/threonine kinase, mitogen-activated protein kinase (MAPK) was a class of protein kinases distributed in cytoplasm with the dual phosphorylation capacity of serine and tyrosine. JNK signal transduction pathway was an important branch of MAPK pathway, which played an important role in various physiological and pathological processes such as cell cycle reproductive apoptosis and cell stress [13]. Previous studies had shown that the JNK signaling pathway modulated inflammation [14, 15]. When triggered by certain stimuli, such as TGF-β1, it caused phosphorylation of JNK. In turn, inhibited JNK increased the production of matrix metalloproteinases 2 and 9 (MMP-2 and 9) [6]. JNK played an important role in inflammatory response by expressing specific proteases and cytokines. Adhesions were caused by excessive inflammation, while tendon gaps and ruptures were caused by insufficient matrix degradation and regeneration due to inflammation [16]. Therefore, inhibition of activation of the JNK pathway may be a promising therapeutic strategy for flexor tendon therapy.

However, the adhesion problem after flexor tendon injury is still not well solved, it is necessary to clarify the pathogenesis and molecular mechanism of tendon tissue after trauma, so as to find a better treatment. In this study, we hypothesized that overexpression of TGF-β3 could reduce the inflammatory response after flexor tendon injury, could reduce adhesion formation, and could enhance the repair strength after tendon injury. To test this hypothesis, the deep flexion tendons of the hind leg in rats models were ruptured and repaired, and the injection of ad-TGF-β3 were initiated in early hyperplasia. Therefore, the aim of the present study was to investigate the inhibitory effect of TGF-β3 on the inflammation and adhesion of flexor tendon repair.

## Materials and methods

### Animal study

All healthy SD male rats (age: 10–12 weeks, weight: 320 ± 20 g) were obtained from the west China Hospital of Sichuan University. Rats were maintained under standard laboratory condition, with free access to food and water and housed prior to experiments in an animal room under standard conditions (23 ± 2 °C; 60 ± 10% humidity; 12 h light/dark cycle). The rats were free of all viral, bacterial, and parasitic pathogens. Experimental animals were not used for breeding purposes. All experiments were performed in accordance with the National Institutes of Health Guide for the Care and Use of Laboratory Animals (NIH Publications No. 8023, revised 1978) and were approved by the Ethical Committee of the west China Hospital of Sichuan University (Chengdu, China).

### Experimental design

Following 1 week of feeding and adaptation, two separate experiments were conducted in this study. Experiment I was designed to assess the expression of TGF-β3 in flexor tendon injury healing model: 30 rats were randomly divided into 5 groups (each *n* = 6), and they were used for RT-qPCR and western blot related experiments at 3, 7, 14, 21 and 28 days after injury repair, respectively. Experiment II was designed to assess the effects of TGF-β3 overexpression on improvement of adhesion after healing of flexor tendon injury in rats: 36 rats were randomly divided into 6 groups (each *n* = 6), of which 18 rats with the vehicle treatment and 18 rats with the TGF-β3 adenovirus vector treatment simultaneously at 14, 21 and 28 days after injury repair, respectively. All the rats were given pentobarbital sodium (Sigma Aldrich; CAS: 57–33-0) to induce general anesthesia, and the towel was routinely sterilized. Incision was made along the side of the middle toe of the posterior claw, and the flexor tendon of the leaky toe was exposed. Then the flexor tendon was cut laterally. After the operation within 1–3 days, there was no braking, and antibiotics were injected to prevent infection. None of the rats in experiment I received treatment. In experiment II, rats in the TGF-β3 group received target-specific injection of TGF-β3 adenovirus vector (ad-TGF-β3, 50 μl of 1 × 10^10 IU/ml) into the tendon repair site from day 3 to 8 after surgery using micro-syringes (1700 series, Hamilton), once daily. The vehicle group received the same amount of vehicle in the corresponding position from day 3 to 8 after surgery, once daily. Then the tendon specimens were collected, and the rats with no treatment were sampled on days 3, 7, 14, 21, and 28, while the rats with TGF-β3 adenovirus vector or vehicle were separately sampled on days 14, 21 and 28, respectively. The blood samples were centrifuged at 3000×g for 10 min at 4 °C and the serum was collected. Blood serum was collected for subsequent hematological or biochemical assays. Death of the rats was verified by the complete cessation of the heartbeat and breathing, and disappearance of reflexes. In addition, flexor tendon tissues of rats treated with no treatment were harvested on post-repair days 3, 7, 14, 21, and 28 for RT-qPCR and western blot, and flexor tendon tissues of rats treated with vehicle or ad-TGF-β3 were harvested on post-repair days 14, 21 and 28 for western blot, adhesion testing, gliding coefficient, biomechanical testing, eosin (H&E) staining, immumohistochemical staining, RT-qPCR, and ELISA (each *n* = 6 rats per treatment per time point).

### Adhesion testing and gliding coefficient

Adhesion tests were performed on days 14, 21, and 28 during flexor tendon healing. The hind leg of the sacrificed rat was immediately dislocated and the flexor tendon was released from the surrounding tissue of the adjacent tarsus. The proximal end of the flexor tendon was fixed between the two bands. The limbs were fixed in a special device, and the shin bones were held firmly in the clamp to prevent rotation with the toes passively extended to a neutral position. Then the neutral position of the metatarsophalangeal joint (MTP) was determined by digital image (zero load). The added weight (0-19 g) was applied to the tendon and a digital photograph was taken for each additional weight. When the angle was normalized to neutra, each image of the metatarsal bone (MTP) bending angles were measured by ImageJ software (http://rsb.info.nih.gov/ij/), and the diagram was drawn with the corresponding load. The gliding resistance was determined by the single-phase exponential equation fitted with the bending data, which the MTP bending Angle = β x (1-exp(−m/α), where m was the applied load (Prism GraphPad 6.0a; GraphPad Software, Inc., San Diego, CA). The curve fit was regulated by the maximum flexion angle (β), for the normal tendons application load was previously determined to be 19 g at 75° [17, 18]. The gliding resistance (α) was determined by non-linear regression, which was an effective method for measuring the buckling resistance of MTP joints due to sliding damage [19]. Besides, the difference in buckling angle between 0 g and 19 g loads was determined as the MTP buckling range of motion (ROM).

### Biomechanical testing

Biomechanical tests were performed on the day 14, 21, and 28 after surgery to evaluate changes in biomechanical properties of the flexor tendon in repair, using the 8841 Instron DynaMight axial servo-hydraulic testing system (Instron Industrial Products, Norwood, MA). The flexor deep toe tendon of the middle toe of the posterior claw was cut off at the metatarsophalangeal joint with a length of nearly 1.5 cm, with the kirschner needle cut through the proximal 1/3 and distal 1/3 lengths of the proximal phalangeal diaphysis, and the kirschner needle tightened on the dorsal side of the phalangeal diaphysis. Clamps were used to fix the kirschner needle on the dorsal side of the phalanges, and the specimen was fixed on the lower clamps. The flexor digitorum profundus tendon was wrapped in thick sandpaper and fixed directly on the upper clamp, and the whole length of the tendon was in the same line. The tension-displacement control test was carried out at the speed of 30 mm/min until failure. The force-displacement data was automatically recorded and plotted to determine the maximum load at failure.

### Histological examination

The cut tendon segments were carefully removed and fixed in 4% (v/v) paraformaldehyde at room temperature for 48 h. The tendon was embedded in paraffin and cut into 4-μm sections. The sections were heated at 60 °C for 1 h and dewaxed with xylene. Following hydration, the sections were stained with 0.5% H&E at room temperature for 5 min, dehydrated with gradient ethanol, cleared with xylene and mounted with neutral gum. Optical microscopy (BA400 Digital, McAldy industrial group co. LTD) was used to examine pathological changes of the flexor tendon tissue (magnification, 100× and 400×). The histological morphology of the tendon and denatured collagen fibers were observed under the light microscope.

### Immumohistochemical staining

The expression of collagen III in the tendon tissue was evaluated using immumohistochemical staining. The tendon segments were embedded in paraffin and sectioned. Then, the paraffin sections were deparaffinized in xylene, rehydrated by ethanol and incubated with 3% hydrogen peroxide. Tendon tissues samples were blocked at room temperature with 3% BSA (Beijing Solarbio Science & Technology Co., Ltd.) and incubated with rabbit anti-collagen III antibody (1:100, cat. no. ab7778; Abcam) at 4 °C overnight. Samples were then washed three times with PBS, treated with horseradish peroxidase goat anti-rabbit IgG secondary antibody (1:2000, cat. no. ab205718; Abcam) for 20 min at 37 °C and rinsed three times with PBS. After incubating with 0.05% 3–3’diaminobenzidine (DAB) substrate buffer solution for 10 min, it was rinsed with distilled water, then redyed and sealed. The positive expression area and strength of collagen type III were observed under a 100× optical microscope.

### RNA extraction and real-time RT-qPCR

Total RNA from individual FDL tendons surrounded by the scar (3, 7, 14, 21, 28 days pre-repair and 14, 21, 28 days post-repair, respectively) was extracted according to TRIzol instructions. 5 μg of total RNA was taken and synthesized into cDNA by reverse transcription reaction, in which the total reaction system was 20 μl. Real-time PCR was run with cDNA, PerfeCTa SYBR Green Super Mix (Quanta Biosciences, Gaithersburg, MD) and gene specific primers for TGF-β3, TGF-β1, TGF-β2, Smad3, Smad7, Mmp9 and Mmp2. All primers were designed by Sangon Biotech (Shanghai) Co., Ltd. (Table 1). The reaction conditions were as follows: 1 cycle at 95 °C for 30 s followed by 40 cycles at 95 °C for 5 s, and 60–66 °C for 30 s. After the reaction, the Ct value of each sample was automatically analyzed by the computer, and the relative mRNA expression was calculated by 2^-ΔΔCt^ with β-actin as the internal control. The specificity of PCR reaction was determined by the melting curve.

**Table 1:** RT-qPCR Primer Sequences

Gene	Forward(5′-3′)	Reverse(5′-3′)
TGF-β3	5′-TGCGCCCCCTCTACATTG-3’	5′-GGTTCGTGGACCCATTTCC-3’
TGF-β1	5′-TGAGTGGCTGTCTTTTGACG-3’	5′-ACTTCCAACCCAGGTCCTTC-3’
TGF-β2	5′-ATGTGCAGGATAATTGCTGCC-3’	5′-TGGTGTTGTACAGGCTGAGG-3’
Smad3	5′-GCAGGCTCTCCAAACCTCT-3’	5′-GTGGAATGTCTCCCCAACTC-3’
Smad7	5′-CTCAAACCAACTGAGACTGTC-3’	5′-AGGCTCCAGAAGAAGTTGGG-3’
Mmp9	5′-GCCGTCTACTC CTCCCCGTG T-3’	5′-GTCTCTCTCCTACCCTCTGG-3’
Mmp2	5′-TCAGTCGATCACTAGCGTCAAT-3’	5′-CTAACTTCTCCCCACAGGGA-3’
β-actin	5′-CACGATGGAGGGGCCGGACTCATC-3’	5′-TAAAGACCTCTATGCCAACACAGT-3’

Forward and reverse primer sequences used for RT-qPCR. Expression levels were normalized to the internal control β-actin, with each sample run in triplicates

### Western blot analysis

Western blot analysis was performed to detect protein expression in individual FDL tendons surrounded by the scar. Protein extraction kit (Pierce, Thermo Fisher, Ltd.) was used to extract protein. The concentration of the sample was determined according to the instructions of KCTMBCA protein quantitative kit. According to the total protein concentration measured, equal amounts of proteins (30 μg) with different molecular weights in the lysate were then separated adopting 10% SDS-PAGE and transferred onto PVDF membrane (Thermo Fisher Scientific, Inc.). Subsequently, the membrane was blocked with 5% skim milk and probed with primary antibodies: mouse anti-TGF-β1 (1:500, ab190503), mouse anti-TGF-β2 (1:1000, ab36495), rabbit anti-TGF-β3 (1:1000, ab36495), rabbit anti-Smad3 (1:1000, ab40854), rabbit anti-Smad7(1:500, ab216428), rabbit anti-c-Jun(1:1000, ab40766), rabbit anti-p-c-Jun(1:1000, ab32385), rabbit anti-JNK(1:1000, ab179461), rabbit anti-p-JNK(1:1000, ab124956). The membrane was incubated with horseradish peroxidase-labeled goat anti-rabbit immunoglobulin G (IgG; 1:2000, ab205718) or goat anti-mouse IgG(1:500, ab150117) at room temperature for 1 h. All antibodies were purchased from Abcam (Cambridge, MA, UK). After X film exposure and development, Bio-Rad automatic gel imaging system was used for imaging preservation. Using ImageJ image analysis software to analyze gray scale, the gray value of the target protein was divided by the internal reference gray value to correct the error. The relative content of target protein was analyzed statistically. β-actin served as an internal reference.

### Determination of inflammatory factors IL-6, TNF-α, COX2 and IL-1β levels in flexor tendon tissues

Levels of inflammatory factors IL-6 (ab100712), TNF-α (ab208348), COX2 (ab210574) and IL-1β (ab197742) were determined by ELISA Kits all from Abcam (Cambridge, MA, UK) according to the manufacturer’s instructions. A blank well and a sample well were set up respectively. The optical density (OD) of each well was measured at 450 nm, and the concentration of inflammatory cytokines was quantified in accordance with the standard curve.

### Statistical analysis

Data were expressed as mean ± standard deviation. The Kolmogorov-Smirnov test was used to assess the normal distribution of variables. SPSS19.0 software (IBM Corp., Armonk, NY, USA) was used to conduct one-way analysis with Dunnett’s post-hoc test or two-way ANOVA with Bonferroni’s post-hoc test. Differences were considered statistically significant at *P* < 0.05 and *P* < 0.01.

## Results

### Low kurtosis occurred in TGF-β3 and Smad7 expression, and high peak appeared in TGF-β1/2 and Smad3 expression during flexor tendon repair from day 14 to day 28

**Fig. 1:**
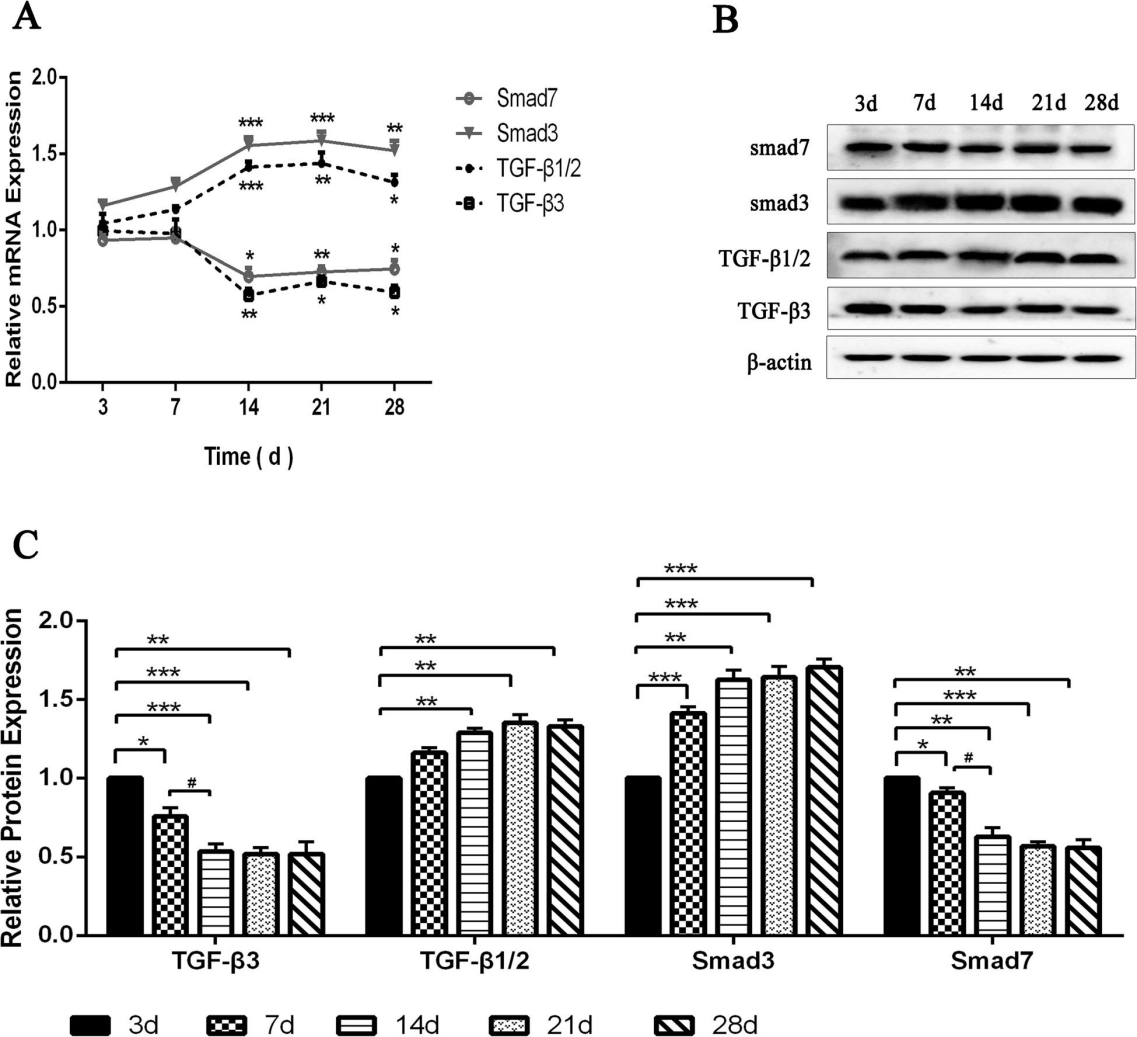
Temporal Expressions of TGF-β3, TGF-β1/2, Smad3, and Smad7 in flexor tendon tissue of post-injury healing models. (**A**) Relative mRNA expressions of TGF-β3, TGF-β1/2, Smad3 and Smad7 in FDL tendon tissues by RT-qPCR. *P < 0.05, **P < 0.01, vs. day 3. (**B**) Protein bands for TGF-β3, TGF-β1/2, Smad3, Smad7 and β-actin by western blotting assay. (**C**) Relative protein expressions of TGF-β3, TGF-β1/2, Smad3 and Smad7 in FDL tendon tissues by western blotting assay. Expression was normalized to β-actin. *P < 0.05, **P < 0.01, ***P < 0.001, vs. day 3; ^#^P < 0.05, ^##^P < 0.01, vs. day 7. *n* = 6 for each group

To investigate the effect of TGF-β3 on the flexor tendon injury healing rat models, the intrasynovial FDL tendon repair models with no treatment were used to determine the expression profile of TGF-β3, TGF-β1/2, Smad3, and Smad7, which would inform the optimum time for ad-TGF-β3 treatment in the study groups. Expressions of TGF-β3, TGF-β1/2, Smad3 and Smad7 in the rat models of flexor tendon injury healing were detected by RT-qPCR and western blot. Relative to the 3rd day, the mRNA expression levels of these four genes in models all showed significant differences from day14 day to day28 (*P* < 0.05), where TGF-β3 and Smad7 showed significantly lower elevations, and TGF-β1/2 and Smad3 showed significantly higher elevations (Fig. 1A). Compared with day 3, TGF-β3 protein showed a significantly reduced change from day 14 to day 28. In order to investigate when the TGF-β3 protein reduction occurred, two adjacent groups were compared in pairs, and it was found that TGF-β3 protein significantly decreased between days 3, 7 and 14, while there was no significant change between day 14 and day 28. Besides, the proteins of TGF-β1/2 and Smad3 both gradually increased from day 3 to day 14. The expression of Smad7 protein showed a significantly downward trend from day 3 to day 14, and a significant downward trend was shown from day 7 to day 14 (Fig. 1B and C).

### TGF-β3 expression was effectively increased by ad-TGF-β3 in the repaired flexor tendon tissue

**Fig. 2:**
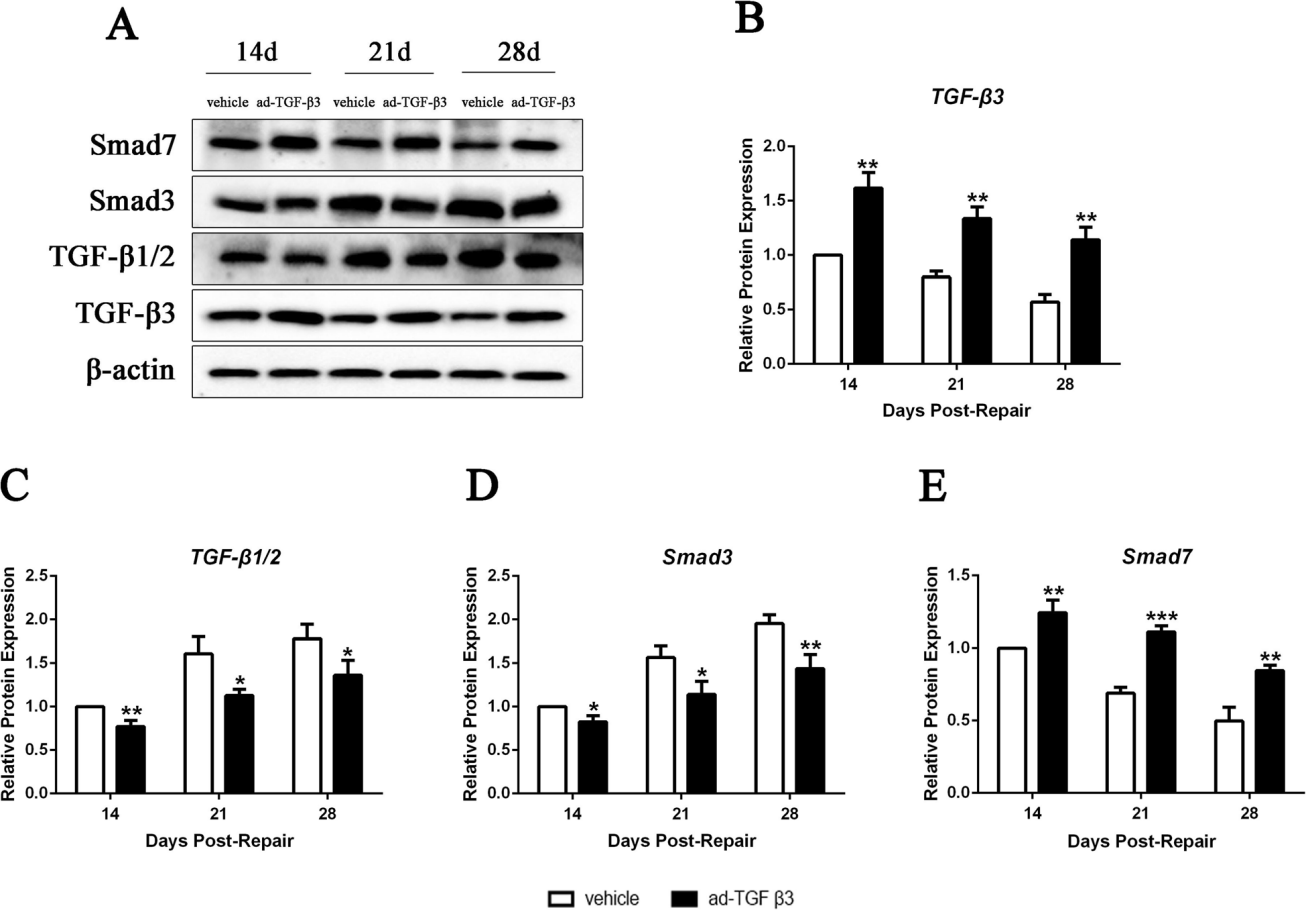
TGF-β3 and Smad7 Expression Increased, and TGF-β1/2 and Smad3 Expression Decreased in ad-TGF-β3 Treated Flexor Tendon Tissues. (**A**) Protein bands for TGF-β3, TGF-β1/2, Smad3, Smad7, and β-actin were detected by western blotting assay. (**B-E**) Relative protein expressions of TGF-β3 (B), TGF-β1/2 (**C**), Smad3 (**D**) and Smad7 (**E**) in flexor tendon tissues were detected by western blotting assay. Expression was normalized to β-actin. *P < 0.05, **P < 0.01, ***P < 0.001 vs. vehicle group. *n* = 6 for each group

In order to investigate the effect of TGF-β3 on adhesion formation in murine model of flexor tendon repair, ad-TGF-β3 was target-specific injected into the tendon repair sites of rats on days 14, 21 and 28, respectively. The ad-TGF-β3 treated rats showed a significant increase trend of TGF-β3 expression in flexor tendon tissues compared to those treated with vehicle on days 14, 21 and 28 post-repair (Fig. 2A and B). On days 14, 21 and 28 post-repair, the TGF-β1/2 expressions and Smad3 expressions in ad-TGF-β3 treated groups were all significantly decreased than those in vehicle groups (Fig. 2 C and D). Furthermore, compared to vehicle groups, there was a significant increase trend of Smad7 expression on days 14, 21 and 28 post-repair (Fig. 2E).

### Ad-TGF-β3 showed an early and transient improvement in gliding function during flexor tendon healing by adhesion testing

**Fig. 3:**
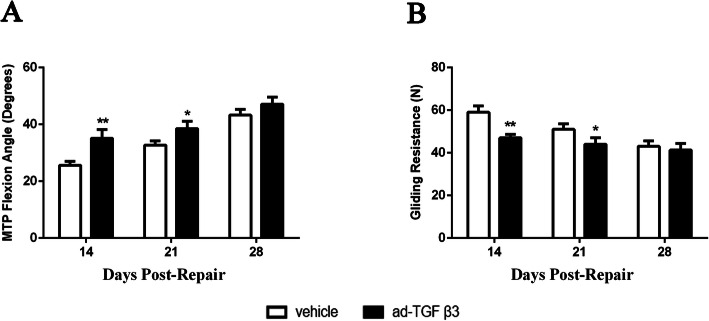
Ad-TGF-β3 Significantly Increased MTP Flexion Angle (**A**) and Decreased Gliding Resistance (**B**) during Flexor Tendon Healing. *P < 0.05, **P < 0.01, vs. vehicle group. *n* = 6 for each group

Significant increase of MTP flexion angle was seen on day 14 and 21 post-repair in ad-TGF-β3 group relative to that in vehicle group, though this significant difference was not shown on day 28 post-repair (Fig. 3A). Besides, rats treated with ad-TGF-β3 had a significantly reduced gliding resistance than that in vehicle groups on day 14 and 21 post-repair, and this difference was disappeared on day 28 (Fig. 3B).

### Ad-TGF-β3 changed mechanical Strenth and stiffness during flexor tendon healing

**Fig. 4:**
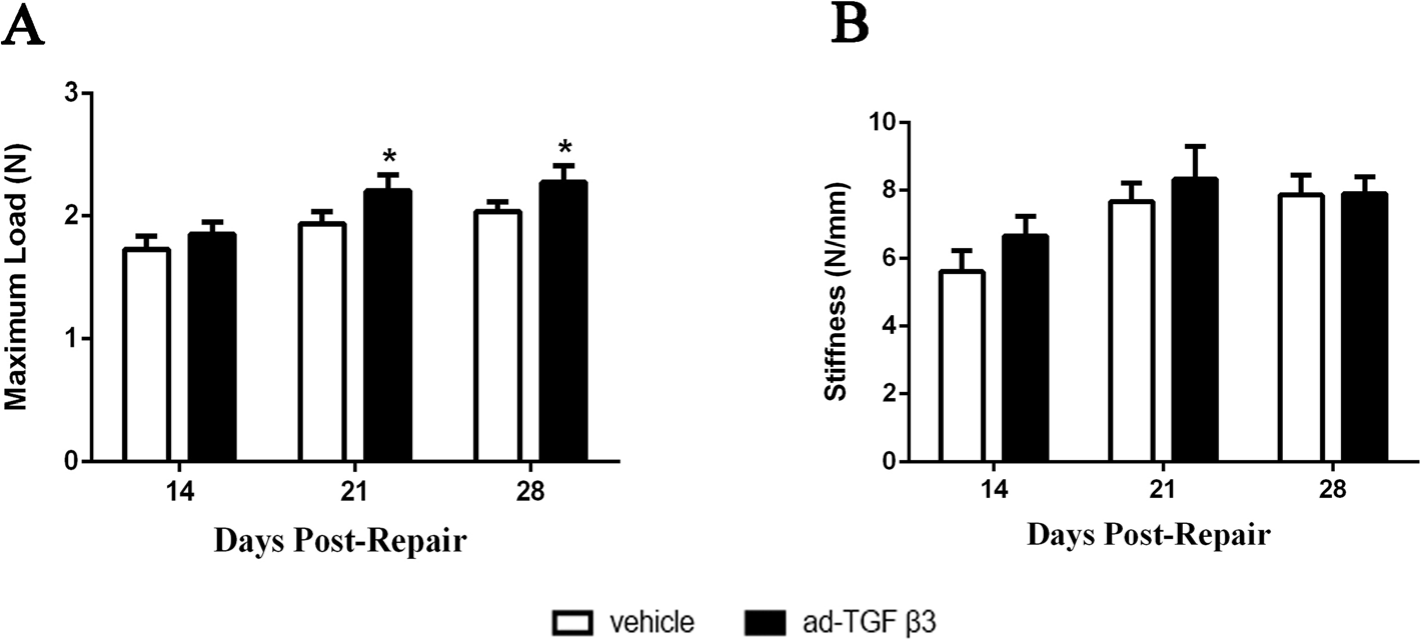
Max Load at Failure and Stiffness during Flexor Tendon Healing. (**A**) Maximum load at failure and (**B**) Stiffness were present between 14 and 28 days during tensile testing, which was no different between vehicle group and ad-TGF-β3 treated group at any given-time. *P < 0.05, vs. vehicle group. *n* = 6 for each group

In terms of the maximum load, there was a significant difference between the vehicle group and the ad-TGF-β3 group on the 21st (vehicle group: 1.93 N ± 0.08 N; ad-TGF-β3 group: 2.20 N ± 0.10 N) and 28th day post-repair (vehicle group: 2.03 N ± 0.07 N; ad-TGF-β3 group: 2.27 N ± 0.11 N), but there was no significant difference between the vehicle group and the ad-TGF-β3 group on the 14th day (vehicle group: 1.73 N ± 0.08 N; ad-TGF-β3 group: 1.85 N ± 0.08 N) (Fig. 4A).

There was no significant difference in the repair stiffness between the vehicle group and ad-TGF-β3 group at any given time. Even so, on day 14 and 21, the stiffness of ad-TGF-β3 group was higher than that in vehicle group (Day14: vehicle group:5.61 N/mm ± 0.50 N/mm, ad-TGF-β3 group: 6.65 N/mm ± 0.47 N/mm; Day21: vehicle group:7.67 N/mm ± 0.45 N/mm, ad-TGF-β3 group: 8.33 N/mm ± 0.78 N/mm), and the stiffness of ad-TGF-β3 group and vehicle were equal on day 28 (Day28: vehicle group:7.86 N/mm ± 0.48 N/mm, ad-TGF-β3 group: 7.90 N/mm ± 0.40 N/mm) (Fig. 4B).

### Ad-TGF-β3 demonstrated a better pathologic recovery process and a significant reduction of collagen type-III expression in the flexor tendon healing tissues

**Fig. 5:**
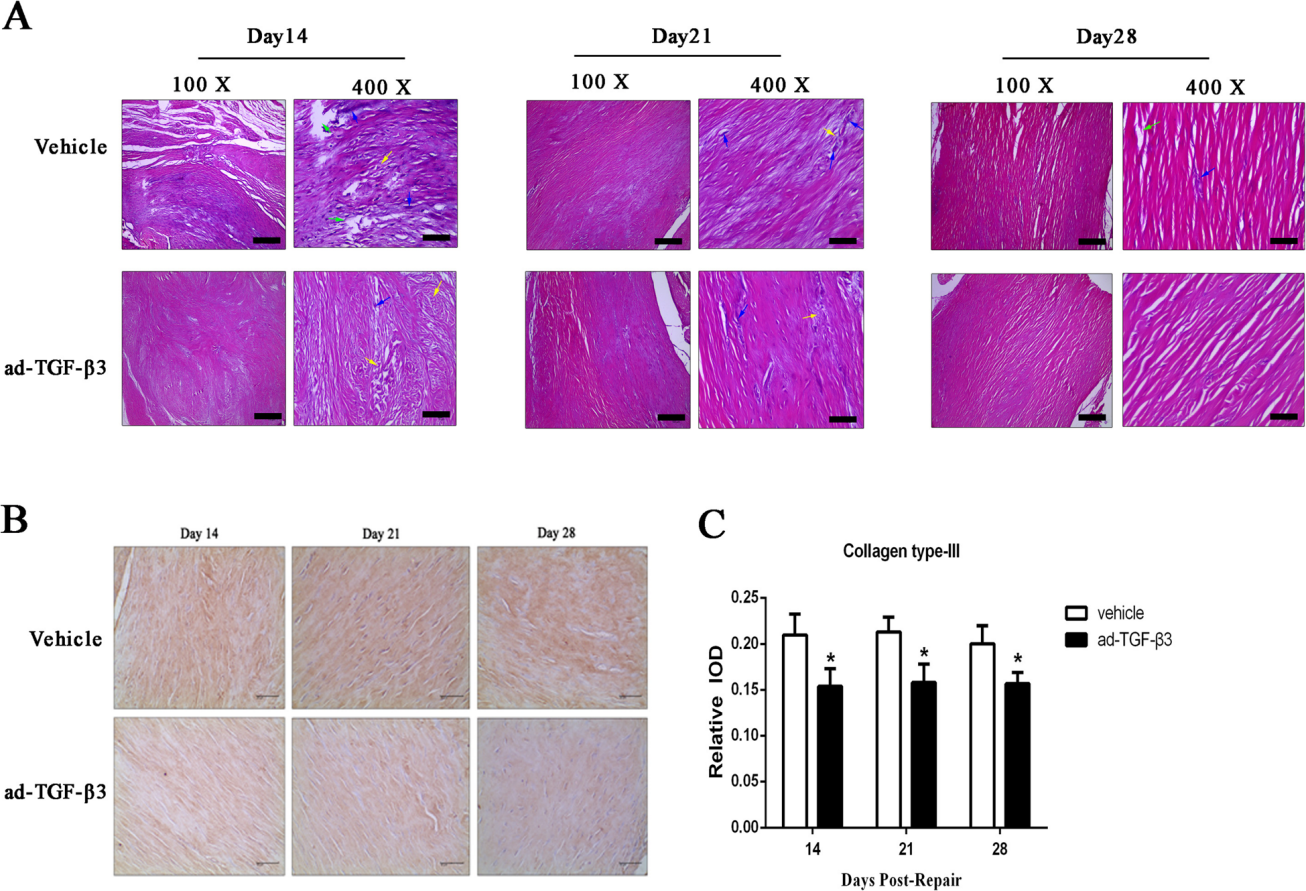
HE Staining and Collagen Type-III Immunohistochemistry Staining. (**A**) HE staining of healing tendon at given days. The yellow arrow indicates the loose and disordered arrangement of collagen fibers, the green arrow indicates the rupture of tendon tissue, and the blue arrow indicates the degeneration and necrosis of collagen fibers. Scale bars = 40 μm. (**B**) Immunohistochemistry of Collagen Type-III in Healing Flexor Tendon Tissues at Day 14, 21, and 28 post-repair. Scale bars = 40 μm. (**C**) Integral optical density (IOD) of Collagen Type-III level showed a significant decrease of ad-TGF-β3 treated group relative to vehicle group in Healing Flexor Tendon Tissues. *P < 0.05, vs. vehicle group. *n* = 6 for each group

HE staining revealed a pathologic process of gradual recovery from day 14 to day 28 in the vehicle and ad-TGF-β3 group. On day 14, vehicle group demonstrated a loose and disordered arrangement of collagen fibers in the tendon tissue, with the rupture or dissolution of the tendon tissue, accompanied by a large amount of degeneration and necrosis of collagen fibers, while in the ad-TGF-β3 group, collagen fibers were arranged in a disordered manner, with part collagen fibers denaturated and necrotic. By day 21, both the vehicle group and the ad-TGF-β3 group were found to have disordered arrangement of collagen fibers in a few areas of tendon tissue, but the amount of collagen fiber degeneration and necrosis in the ad-TGF-β3 group was less than that in the vehicle group. By the 28th day, collagen fibers in tendon tissues in both the vehicle group and the ad-TGF-β3 group were neatly arranged, and a small amount of collagen fibers in the vehicle group were occasionally necrotic and fractured, while the tendon tissues in the ad-TGF-β3 group were intact and clear, with uniform cytoplasmic staining, and no obvious necrosis or fracture of collagen fibers (Fig. 5A).

Immunohistochemistry staining showed the expression of Collagen Type-III was lower in ad-TGF-β3 treated group than that in the vehicle group on day 14, 21, and 28 (Fig. 5B). Semiquanttative analysis revealed that the relative IOD was significantly reduced in ad-TGF-β3 treated group compared to that in vehicle group on day 14, 21, and 28 post-repair (Fig .5C).

### Ad-TGF-β3 increased the mRNA expression levels of Mmp9 and Mmp2 in the flexor tendon healing tissues

**Fig. 6:**
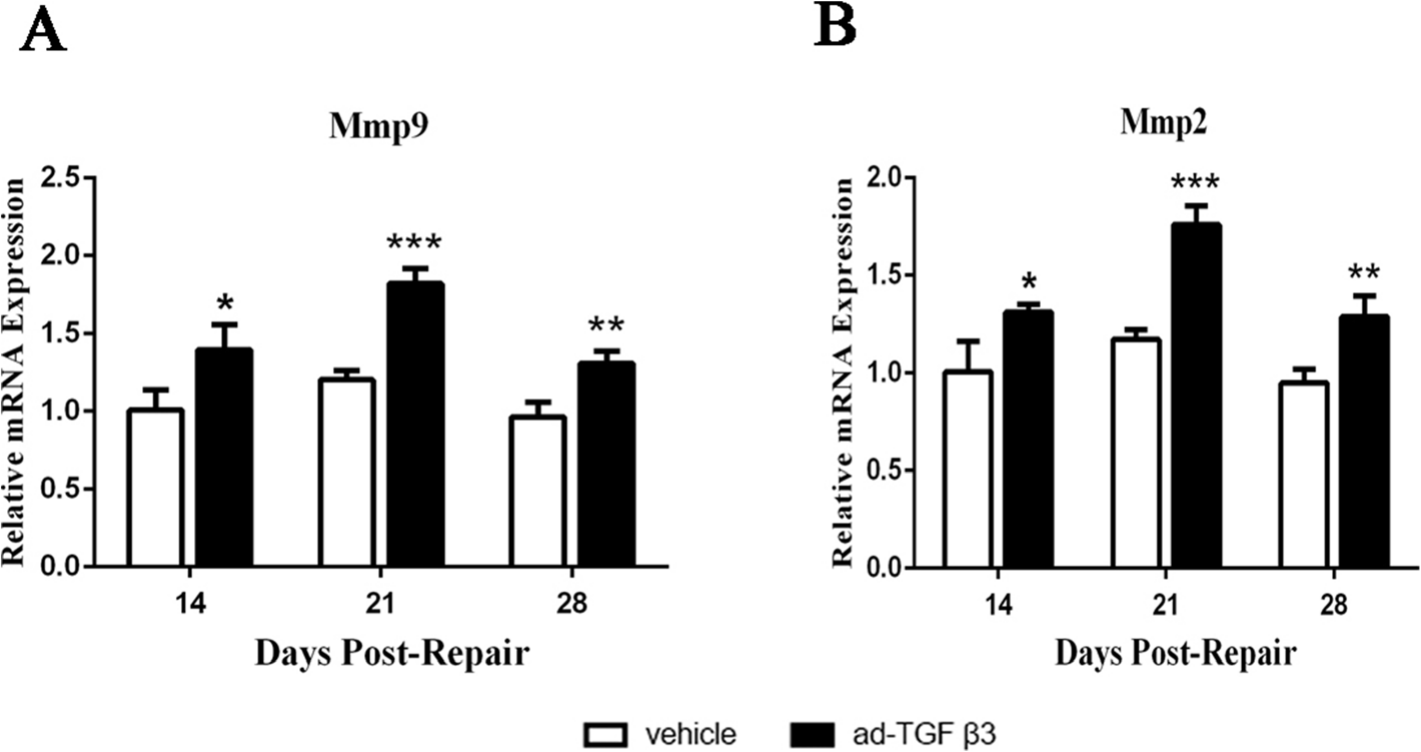
Mmp9 and Mmp2 mRNA Expression Decreased in Ad-TGF-β3 Treated Repairs by RT-qPCR. (**A**) Relative mRNA Expression of Mmp9 (**A**) and Mmp2 (**B**) in flexor tendon tissues. Expression was normalized to β-actin.. *P < 0.05, **P < 0.01, ***P < 0.001 vs. vehicle group. *n* = 6 for each group

In this study, RT-qPCR was used to investigate the effect of ad-TGF-β3 on Mmp9 and Mmp2 expression during flexor tendon healing. Expressions of Mmp9 and Mmp2 were significantly decreased in the ad-TGF-β3 group relative to those in vehicle group on 14, 21, and 28 days post-repair (Fig. 6A and B).

### Ad-TGF-β3 improved inflammation of the flexor tendon healing tissues

**Fig. 7:**
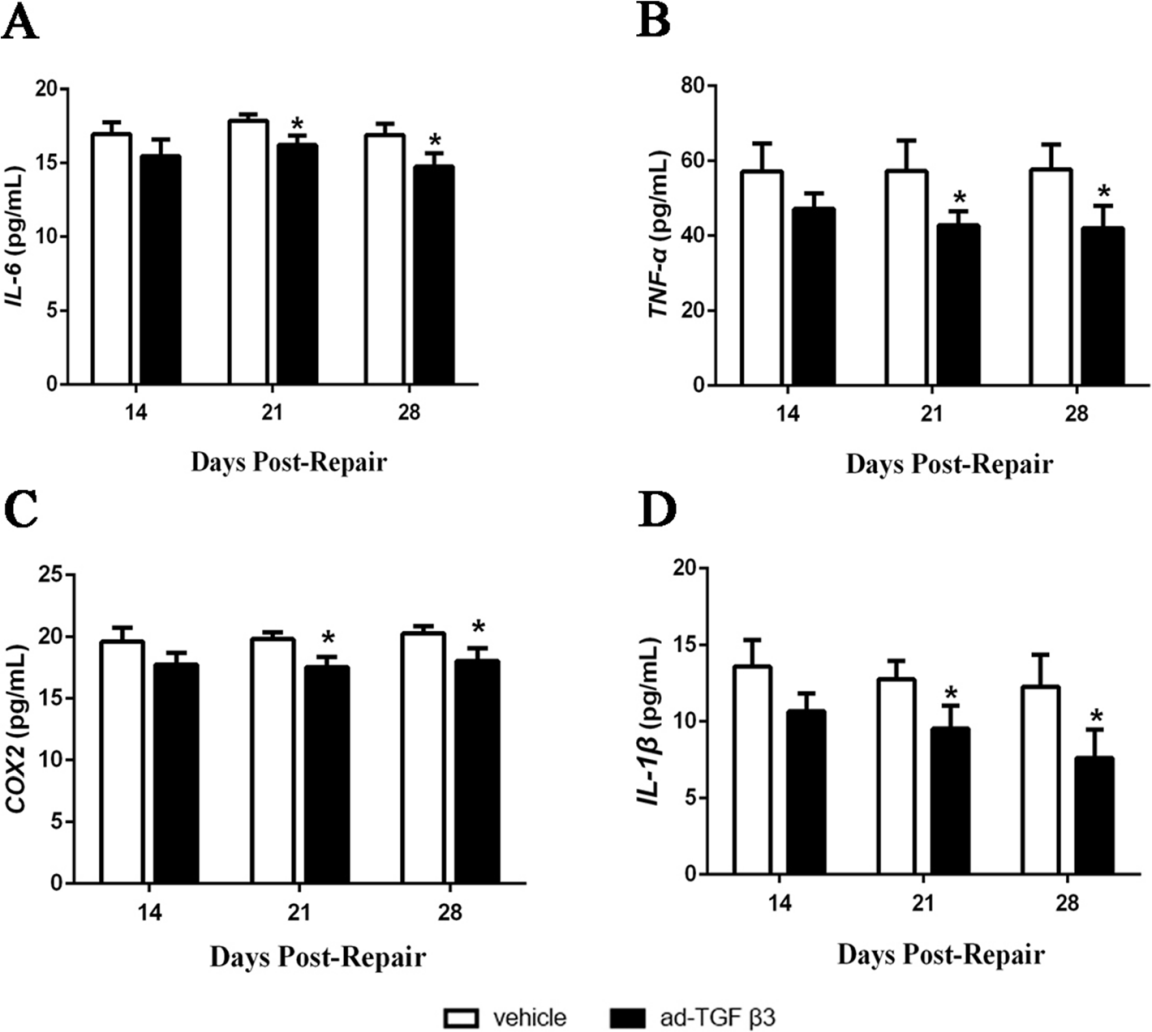
Ad-TGF-β3 Reduced the Inflammatory Factors IL-6, TNF-α, COX2, and IL-1β during Flexor Tendon Healing. Concentration of IL-6 (**A**), TNF-α (**B**), COX2 (**C**), and IL-1β (**D**) in the flexor tendon healing tissues at given-days. *P < 0.05, vs. vehicle group. *n* = 6 for each group

Levels of inflammatory factors were determined by ELISA Kits. Compared to vehicle group, the IL-6 (Fig. 7A), TNF-α (Fig. 7B), COX2 (Fig. 7C) and IL-1β (Fig. 7D) cytokines of the healing flexor tendon tissues in ad-TGF-β3 group were all significantly reduced at day 21 and 28 post-repair, and these were accompanied by no significant difference on day 14 post-repair.

### Ad-TGF-β3 inhibited the JNK/c-Jun signaling pathway in rats models during flexor tendon healing

**Fig. 8:**
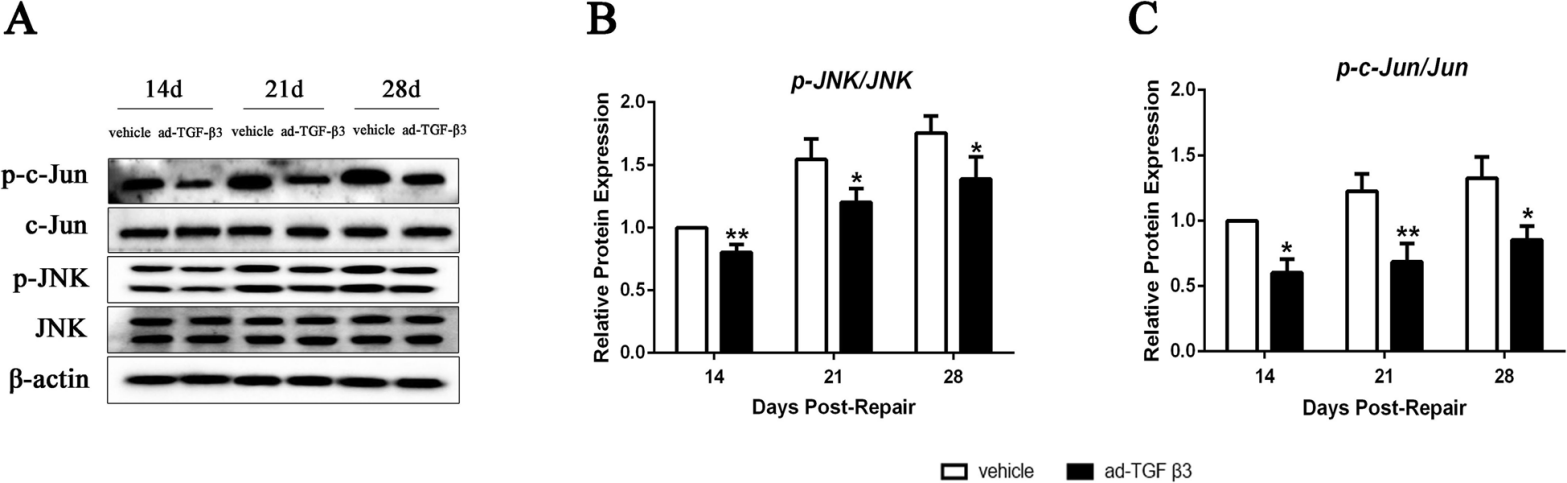
Ad-TGF-β3 Reduced JNK/c-Jun Signaling Pathway during Flexor Tendon Healing. (**A**) Protein bands for JNK, p-JNK, c-Jun, p-c-Jun and β-actin by western blotting assay. (B,C) Relative protein expression of p-JNK/JNK (**B**) and p-c-Jun/Jun(**C**). *P < 0.05, **P < 0.01, vs. vehicle group. *n* = 6 for each group

Western blot was used to investigate whether ad-TGF-β3 has an effect on JNK/c-Jun pathway during Flexor Tendon Healing. Compared with the vehicle group, the ratio of p-JNK/JNK decreased significantly in ad-TGF-β3 group on day 14, 21 and 28 post-repair (Fig. 8A and B). Additionally, the ratio of c-Jun/p-c-Jun reduced significantly in ad-TGF-β3 group compared to those in vehicle group on day 14, 21 and 28 post-repair (Fig. 8C).

## Discussion

Tendon adhesion refers to the proliferation and invasion of the surrounding tissues and the inflammatory reaction in the process of tendon healing, which can lead to tendon motor dysfunction. In this study we examined the anti-adhesion, anti-inflammatory and JNK/c-Jun pathway effects of TGF-β3 on the of flexor tendon repair models.

TGF-β3 has been shown to exert its biological activity on the healing process of tendon cell injury by down-regulating Smad3 levels and up-regulating Smad7 levels in tendon cells [12]. TGF-β3, as an isomer of TGF-β1 in vivo, can down-regulate the levels of TGF-β1 and TGF-β2, which plays the role of TGF-β1 antibody, to inhibit scar formation [20, 21]. Therefore, quantifying TGF-β3, TGF-β1/2, Smad3, and Smad7 in the repair sites could investigate whether TGF-β3 was effectively expressed. Multiple studies have found high levels of TGF-β3 and low levels of TGF-β1 and TGF-β2 during scar free healing in embryonic tissue after trauma, whereas in adults scar healing, the levels of TGF-β3 are lower than that of TGF-β1 and TGF-β2 [20, 21]. In the preliminary experiment, we further studied the regulation of Smad3 and Smad7 proteins in the TGFβ/Smad signaling pathway of tendon cells [12]. It was found that the addition of TGF-β3 in the process of tendon cell injury healing could down-regulate the expression of Smad3 protein and up-regulate the expression of Smad7 protein, which preliminarily elucidated the possible regulatory mechanism of TGF-β3 in promoting scar free tendon healing, suggesting that TGF-β3 may reduce tendon adhesion [12]. Given the closely connection between TGF-β3 and scar formation [12], we tested the hypothesis that ad-TGF-β3 would reduce adhesion formation and improve tendon gliding function. Our data demonstrated that, after the operation within 1–3 days, antibiotic injection acted as a sterile infection. Then TGF-β3 and Smad7 expressions declined significantly faster in rats with no treatment, while they increased significantly after treating with ad-TGF-β3 from the 14th day. Also from the 14th day, TGF-β1/2 and smad3 expressions significantly increased fastest with no treatment, while decreased significantly in the ad-TGF-β3 treated group. Such cases were all consistent with the results that ad-TGF-β3 improved tendon gliding function during flexor tendon healing at 14 days post-repair. This study showed that TGF-β3 up-regulated MTP flexion angle of FDL tendon, and down-regulated the gliding resistance from day 14. Though there were no significant differences in the results of repair stiffness between the vehicle group and ad-TGF-β3 group at any given time, a higher max load and a harder stiffness were displayed with the increase of time, and the max load in the ad-TGF-β3 group was relatively higher than that in the vehicle group. These mechanical experiments intuitively demonstrated the improvement effects of TGF-β3 on flexor tendon healing.

Whether TGF-β3 improving the pathological features of recovering tendon tissue was still lacking direct evidence. To this end, we started from the pathological study of flexor tendon tissues in healing, observed the histopathological features of the tendon, analyzed its influence on the secretion of type III collagen, and discussed the relationship between TGF-β3 and flexor tendon adhesion. Tendon was the dense connective tissue connecting muscle and bone, which was rich in collagen, mainly collagen I, and a small amount of type collagen III [22]. The characteristics of collagen matrix of tendon largely reflected the characteristics of tendon, and the pathological changes of tendon were mainly depended on the changes of collagen matrix [23–25]. Studies had shown that higher levels of stromal remodeling were common in ruptured tendons, and the amount of collagen in tendon disease animal model decreased significantly, but collagen type-III percentage increased relative to normal tissues [26]. Furthermore, the main reason for the pathological changes in tendon tissue was the change of collagen matrix, which led to the interruption of collagen fiber bundle continuity, resulting in the loose structure and fractures [23, 25]. In this study, the transverse severance of flexor deep tendon ultimately led to the imbalance of the homeostasis of the tendon tissue, which affected the synthesis of the extracellular matrix and resulted in tendon degeneration [27, 28]. The results showed that, under the treatment of ad-TGF-β3 during flexor tendon tissue healing, a better pathologic recovery process was seen by HE staining. Besides, type III collagen secretion were significant decreased, indicating that ad-TGF-β3 reduced the synthesis and secretion of collagen III associated with injury repair, suggesting that ad-TGF-β3 reduced tendon degeneration, which provided histological evidence for the inflammatory mediators theory of tendinopathy mechanism.

Tendon adhesion was closely related to tendon healing. Previous studies had shown that MMPs were associated with tendon injury [29, 30]. The net effect of increased MMP activity was matrix degradation, which became part of the remodeling process in wound healing [31]. Besides, Mmp9 was involved in collagen degradation, and Mmp2 was not only involved in collagen degradation, but also in collagen remodeling, which were closely related to adhesion formation [32]. Mmp2 and Mmp9 could degrade many small tendon fragments, and some MMPs such as Mmp2 mediated the healing process [29, 30]. In this study, the inflammations of tendon tissue were aggravated, and the degradations of extracellular matrix were increased, resulting in tendon injury. Therefore, TGF-β3 played an important role in tendon adhesion by decreasing the secretion of Mmp9 and Mmp2. Other studies had shown that Mmp9 could lead to tendon adhesion by comparing the repair model of tendon injury between Mmp9 gene deletion rats and non-gene deletion rats [33]. On day 14, 21 and 28 after administration, ad-TGF-β3 were found to have significant inhibitory effects on Mmp9 and Mmp2. Some studies indicate that Mmp9 was a potential target to limit adhesion formation in tendon healing [18]. In this study, a significant reduction of Mmp9 was maintained at any given time in the tendon tissue under the stimulation of ad-TGF-β3. These results indicated that ad-TGF-β3 inhibited not only collagen degeneration involved in Mmp9, but also collagen degeneration and remodeling involved in Mmp2.

The strength of the tendon itself mainly depended on the endogenous repair mechanism, while the exogenous repair mainly formed adhesion [23, 25]. Research on the mechanism of tendon injury showed that excessive load led to the injury of tendon fibers, which would release a large number of inflammatory factors [28]. With the continuous accumulation of inflammatory and collagen stimuli, the healing flexor tendon tissues secreted inflammatory cytokines such as IL-6, TNF-α, COX2, and IL-1β, and these cytokines promoted the accumulation of fibrin, and then led to the formation of fiber adhesion [34, 35]. Inflammatory cytokines played a significant role in the process of tendon repair [23, 35]. This study suggested that TGF-β3 prevented tendon adhesion by reducing local inflammatory response.

TGF-β3 activated the JNK signal, which played an important role in inflammatory response by expressing specific proteases and cytokines [14, 15]. In order to further explore the anti-inflammatory molecular mechanism of TGF-β3 on adhesion of flexor tendon, the roles of inflammation-related JNK/c-Jun signaling pathway were investigated. JNK signal transduction pathway played an important role in various physiological and pathological processes such as cell cycle reproductive apoptosis and cell stress [13]. TGF-β could activate the JNK signal, which expressed specific proteases and cytokines, thereby playing an important role in inflammatory response [14, 15]. Therefore, TGF-β3 might carry out meaningful activities by attenuating inflammatory responses through down-regulation of the JNK/c-Jun pathway. In this present study, p-JNK and p-c-Jun levels in the flexor tendon tissues during healing were markedly decreased by ad-TGF-β3 treatment, suggesting that the anti-inflammatory effect of TGF-β3 may occur by inhibiting the JNK/c-Jun signaling pathway.

In conclusion, the present study successfully demonstrated the protective effect of TGF-β3 against flexor tendon damage. The possible mechanisms underlying the protective effect of flexor tendon damage might be associated with the improvement of adhesion, anti-inflammatory effect and inhibition of JNK/c-Jun pathway. The present findings had explored the regulatory mechanism of TGF-β3 during flexor tendon healing, which provided a new idea for the prevention and treatment of scar adhesion after tendon injury.

## Data Availability

The datasets used during the present study are available from the corresponding author on reasonable request.

## References

[1] Loiselle AE, Kelly M, Hammert WC. Biological augmentation of flexor tendon repair: a challenging cellular landscape. J Hand Surg Am. 2015:144–9. 10.1016/j.jhsa.2015.07.002 26652792

[2] Wong JK, Lui YH, Kapacee Z, Kadler KE, Ferguson MW, DA MG (2009). The cellular biology of flexor tendon adhesion formation: An old problem in a new paradigm. Am J Pathol.

[3] Harrison RK, Mudera V, Grobbelaar AO, Jones ME, DA MG (2003). Synovial sheath cell migratory response to flexor tendon injury: an experimental study in rats. J Hand Surg.

[4] Okamura T, Morita K, Iwasaki Y, Inoue M, Komai T, Fujio K, Yamamoto K (2015). Role of TGF-beta3 in the regulation of immune responses. Clin Exp Rheumatol.

[5] Gilbert RWD, Vickaryous MK, Viloria-Petit AM (2021). Signalling by transforming growth factor Beta isoforms in wound healing and tissue regeneration. J Dev Biol.

[6] Hou TY, Wu SB, Kau HC, Tsai CC (2021). JNK and p38 inhibitors prevent transforming growth factor-β1-induced Myofibroblast Transdifferentiation in human Graves’ orbital fibroblasts. Int J Mol Sci.

[7] Cheifetz S, Hernandez H, Laiho M, ten Dijke P, Iwata KK, Massagué J (1990). Distinct transforming growth factor-beta (TGF-beta) receptor subsets as determinants of cellular responsiveness to three TGF-beta isoforms. J Biol Chem.

[8] Kohama K, Nonaka K, Hosokawa R, Shum L, Ohishi M (2002). TGF-beta-3 promotes scarless repair of cleft lip in mouse fetuses. J Dent Res.

[9] Hakvoort T, Altun V, van Zuijlen PP, de Boer WI, van Schadewij WA, van der Kwast TH (2000). Transforming growth factor-beta (1), −beta (2), −beta (3), basic fibroblast growth factor and vascular endothelial growth factor expression in keratinocytes of burn scars. Eur Cytokine Netw.

[10] Beredjiklian PK, Favata M, Cartmell JS, Flanagan CL, Crombleholme TM, Soslowsky LJ (2003). Regenerative versus reparative healing in tendon: a study of biomechanical and histological properties in fetal sheep. Ann Biomed Eng.

[11] Kuo CK, Petersen BC, Tuan RS (2008). Spatiotemporal protein distribution of TGF-betas, their receptors, and extracellular matrix molecules during embryonic tendon development. Dev Dyn.

[12] Jiang K, Chun G, Wang Z, Du Q, Wang A, Xiong Y (2016). Effect of transforming growth factor-beta3 on the expression of Smad3 and Smad7 in tenocytes. Mol Med Rep.

[13] Zhou YY, Li Y, Jiang WQ, Zhou LF (2015). MAPK/JNK signalling: a potential autophagy regulation pathway. Biosci Rep.

[14] Wang JQ, Xu ZH, Liang WZ, He JT, Cui Y, Liu HY, Xue LX, Shi W, Shao YK, Mang J (2016). Effects of c-Jun N-terminal kinase on Activin A/Smads signaling in PC12 cell suffered from oxygen-glucose deprivation. Cell Mol Biol (Noisy-le-grand).

[15] Suchal K, Malik S, Gamad N, Malhotra RK, Goyal SN, Ojha S, Kumari S, Bhatia J, Arya DS (2016). Mangiferin protect myocardial insults through modulation of MAPK/TGF-beta pathways. Eur J Pharmacol.

[16] Shen H, Jayaram R, Yoneda S, Linderman SW, Sakiyama-Elbert SE, Xia Y, Gelberman RH, Thomopoulos S (2018). The effect of adipose-derived stem cell sheets and CTGF on early flexor tendon healing in a canine model. Sci Rep.

[17] Loiselle AE, Bragdon GA, Jacobson JA, Hasslund S, Cortes ZE, Schwarz EM, Mitten DJ, Awad HA, O’Keefe RJ (2009). Remodeling of murine intrasynovial tendon adhesions following injury: MMP and neotendon gene expression. J Orthop Res.

[18] Loiselle AE, Frisch BJ, Wolenski M, Wolenski M, Jacobson JA, Calvi LM, Schwarz EM, Awad HA, O’Keefe RJ (2012). Bone marrow-derived matrix metalloproteinase-9 is associated with fibrous adhesion formation after murine flexor tendon injury. PLoS One.

[19] Hasslund S, Jacobson JA, Dadali T, Basile P, Ulrich-Vinther M, Søballe K, Schwarz EM, O’Keefe RJ, Mitten DJ, Awad HA (2008). Adhesions in a murine flexor tendon graft model: autograft versus allograft reconstruction. J Orthop Res.

[20] Klass BR, Rolfe KJ, Grobbelaar AO (2009). In vitro flexor tendon cell response to TGF-beta1: a gene expression study. J Hand Surg Am.

[21] Bullard KM, Longaker MT, Lorenz HP (2003). Fetal wound healing: current biology. World J Surg.

[22] Magnusson SP, Narici MV, Maganaris CN, Kjaer M (2008). Human tendon behaviour and adaptation, in vivo. J Physiol.

[23] Walz DM, Newman JS, Konin GP (2010). Epicondylitis: pathogenesis, imaging, and treatment. Radiographics.

[24] Sullo A, Maffulli N, Capasso G, Testa V (2001). The effects of prolonged peritendinous administration of PGE1 to the rat Achilles tendon: a possible animal model of chronic Achilles tendinopathy. J Orthop Sci.

[25] Fredberg U, Stengaard-Pedersen K (2008). Chronic tendinopathy tissue pathology, pain mechanisms, and etiology with a special focus on inflammation. Scand J Med Sci Sports.

[26] Lui PY, Chan LS, Lee YW, Fu SC, Chan KM (2010). Sustained expression of proteoglycans and collagen type III/type I ratio in a calcified tendinopathy model. Rheumatology.

[27] Buono AD, Battery L, Denaro V, Maccauro G, Maffulli N (2011). Tendinopathy and inflammation: some truths. Int J Immunopathol Pharmacol.

[28] Battery L, Maffulli N (2011). Inflammation in overuse tendon injuries. Sports Med Arthrosc.

[29] Maffulli N, Oliva F, Del Buono A, Osti L. Metalloproteases and tendinopathy. 2013;3:51–7. 10.1007/978-1-4471-4103-7_4. 10.11138/mltj/2013.3.1.051 PMC3676164 23885345

[30] Visse R, Nagase H (2003). Matrix metalloproteinases and tissue inhibitors of metalloproteinases. Circ Res.

[31] Riley GP, Curry V, Degroot J, El BV VN, Hazleman BL, Bank A (2002). Matrix metalloproteinase activities and their relationship with collagen remodelling in tendon pathology. Matrix Biol.

[32] Oshiro W, Xing XY, Tu YZ, Manske PR (2003). Flexor tendon healingin the rat:a histologic and gene expression study. J Hand Surg.

[33] Loiselle AE, Frisch BJ, Wolenski M, Jacobson JA, Calvi LM, Schwarz EM, Awad HA, O’Keefe RJ (2012). Bone marrow-derived matrix metalloproteinase-9 is associated with fibrous adhesion formation after marine flexor tendon injury. PLoS One.

[34] Edsfeldt S, Holm B, Mahlapuu M, Reno C, Wiig M (2016). PXL01 in sodium hyaluronate results in increased PRG4 expression: a potential mechanism for anti-adhesion. Ups J Med Sci.

[35] Riley G (2008). Tendinopathy-from basic science to treatment. Nat Clin Pract Rheumatol.

